# Robotic assisted orbital surgery for resection of advanced periocular tumours – a case series report on the feasibility, safety and outcome

**DOI:** 10.1038/s41433-024-02932-6

**Published:** 2024-02-22

**Authors:** Mohsan Malik, Claire Daniel, Jack Faulkner, Jimmy Uddin, Asit Arora, Jean-Pierre Jeannon

**Affiliations:** 1 Adnexal Service, Moorfields Eye Hospital, London, EC1V 2PD UK; 2 Head and Neck Oncology, Guys and St Thomas Hospital, London, SE1 9RT UK

**Keywords:** Technology, Eye cancer, Surgery, Outcomes research

## Abstract

**Purpose:**

Orbital surgery benefits from well-designed instrumentation that offers gentle tissue manipulation, high manoeuvrability and control. Nevertheless, in confined spaces, tissue manipulation must be accomplished with exceptionally high accuracy and precision. This is where robotic surgery offers an advantage. We aimed to evaluate a robotic-assisted surgical system’s feasibility, safety and outcome in assisting tumour clearance.

**Patients and methods:**

A case series of patients with advanced periocular tumours undergoing robotic-assisted globe-sparing resection was performed using the DaVinci XI system (Intuitive Surgical, Inc). Institutional ethics and multidisciplinary approval were sought in all cases.

**Results:**

Four patients with advanced periocular tumours underwent robotic-assisted orbital surgery at a mean age of 63 years (range 42–86). Two patients were diagnosed with squamous cell carcinoma, and two had basal cell carcinoma. One patient was found to have positive lymph nodes at the time of surgery and underwent simultaneous parotidectomy and lymph node clearance. Clear resection of the primary tumour was achieved in all patients; three patients underwent further resection due to narrow margins prior to reconstruction. Patients were follow-up for at least one year, and three remained disease-free. One patient with pre-existing extra-orbital disease developed metastatic disease four months post-op. All patients preserved vision peri-operatively, with no complaints of diplopia. Moderate ocular surface disease was noted in two patients.

**Conclusion:**

Our series highlights the potential advantage of three-dimensional optics, multi-directional instrumentation and motion scaling technology to achieve globe-sparing tumour resection in advanced periocular tumours. However, further robotic instrumentation development is required for orbital surgery.

## Introduction

Numerous surgical fields have adopted robotic systems for increased precision, manoeuvrability, and improved visualisation over manual surgery. Since its introduction 20 years ago, the DaVinci Surgical System (Intuitive Surgical, Inc.) has become the most prevalent clinically approved robotic-assisted surgical system, transforming care in gynaecology, urology and general surgery [1]. Randomised controlled trials have demonstrated that robotic-assisted procedures have comparable clinical outcomes with possible improved quality of life, quicker recovery and reduced post-operative pain scores in some patient groups [2].

While robotic systems have made rapid progress in allied surgical fields such as urology, thoracic and gynaecology, they have yet to be implemented into oculoplastic and adnexal surgery. Orbital surgery presents a unique challenge for surgical innovation: the orbit is a highly vascularised, cone-shaped cavity filled with the globe and closely related to the skull base and paranasal sinuses. The dimensions of the orbit are significantly smaller compared to the abdominal or thoracic cavity, hence increasing the complexity and challenge to surgically engineer.

Traditional curative surgical approaches to orbital pathology have been limited to remodelling bony walls (decompression) and open surgical resection of lesions, limiting the risk of haemorrhage. Moreover, given the anatomical relations and the presence of optic nerve and vasculature, posterior (orbital apex) dissection or use of monopolar cautery has been used with extreme caution to prevent injury– directly through trauma or indirectly through electro-thermal necrosis [3]. Robotic-assisted surgical devices may overcome these issues through scaled motion which facilitate a high degree of precision in movement and enhanced visualisation in deep cavity.

We recently reported the world’s first in man robotic-assisted orbital surgery for the resection of advanced periocular tumours [4], demonstrating preserved ocular tissue with no immediate adverse complications; however, long-term outcomes and safety were not reported. We aimed to evaluate the feasibility, 12-month safety and outcomes of tumour clearance using a robotic-assisted surgical system.

## Methods

### Robotic assistive device and development

**Fig. 1:**
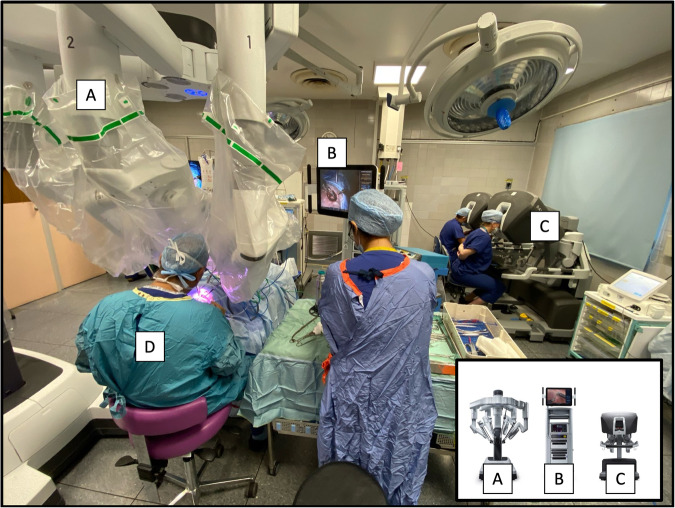
Operating Room Set-up and DaVinci XI Robotic Surgical System [20]. **A** Patient Cart - octopus design multi-arm robot-assisted surgical device holding the endoscopic camera and instruments which is positioned above the patient’s eye **B** Vision Cart supports the 3D HD endoscopic system, **C** Surgeon Console - facilitates remote manipulation of surgical device with high-definition 3D viewing system for primary surgeon and assistant if required, **D** Bedside surgeon and surgical scrub team.

All cases in our series were performed using DaVinci XI system (Intuitive Surgical, Inc). It consists of two components; a remote console to visualise and operate the robotic surgical unit, and an operating unit with two multi-articulated arms with changeable tools and a three-dimensional endoscope with up to 10 times magnification and autofocus (Fig. 1).

Pre-operative robotic device set up, positioning and tool selection was performed in a dry and wet lab to optimise configuration. The final surgical tools configured with left-hand permanent cautery spatula to incise tissue and right-hand Maryland bipolar forceps for haemostasis and tissue manipulation. Moreover, the motion was scaled by a factor of three to facilitate fine delicate manipulation and dissection of tissue.

This study had Institutional Review Board approval and adhered to the tenets of the Declaration of Helsinki. Furthermore, given the novelty of the procedure performed, Trust Risk and Assurance Committee (TRAC) and Robotic Steering Group (RSG) approval was sought before performing the procedures. Patients were informed with enhanced patient information leaflet and consent.

### Patient selection

Patients with advanced periocular eyelid tumours were discussed in the head and neck multidisciplinary team meeting, composed of otolaryngologists with a specialist interest in head and neck oncology and robotic surgery, adnexal surgeons, oncologists, radiologists and pathologists. Patients were offered robotic-assisted surgical resection of tumours as an alternative to orbital exenteration for tumour clearance.

### Robotic assistive procedure

The area to be excised was marked pre-operatively with a 5–10 mm margin dependent on the underlying pathology and tissue excised en-bloc. A bedside surgeon was required to assist with the retraction of soft tissue and globe, as well as suction. A non-robotic piezoelectric saw was used if osteotomy was required. Reconstruction was staged, pending pathological clearance.

### Outcome measures

Tumour clearance and 12-month survival were assessed. We also assessed post-operative visual function and complications associated with the procedure.

## Results

Four patients with advanced periocular tumours were included in the feasibility study. Four patients with advanced periocular tumours underwent robotic-assisted orbital surgery at a mean age of 63 years (range 42–86) (Table 1).

**Table 1:** Patient demographics and outcomes.

Patient Number	Age	Sex	Diagnosis (Stage)	Follow-up	Status	Pre-op VA	Post-op VA
1	86	F	BCC (T3N0M0)	26	DF	20/60	20/60
2	52	M	BCC (T2N0M0)	18	DF	20/30	20/30
3	70	M	SCC (T2N0M0)	18	DF	20/15	20/15
4	42	M	SCC (T3N1M0)	12	DC	20/20	20/80

*BCC* Basal cell carcinoma, *SCC* squamous cell carcinoma, *DF* disease free, *DC* disease continued, *VA* visual acuity.

Patient one; an 86-year-old female with a recurrence of basal cell carcinoma fifteen years post-Mohs micrographic surgery. The 2.2 cm mass from the right medial canthus was invading the anterior medial orbit encompassing the nasolacrimal apparatus—no evident involvement of the lacrimal drainage, bone, perineural spread or lymphadenopathy on pre-operative imaging. The patient underwent en-bloc robotic-assisted resection of the tumour with manual osteotomy of the adjacent frontal process of the maxilla, nasal and lacrimal bone. Histology demonstrated complete resection of 16 mm basosquamous carcinoma with perineural invasion, without bone involvement, with a close margin inferior-medially. The patient went under further resection at the time of reconstruction with a radial forearm free flap.

The patient remains disease-free 26 months post-operative. Patient reported no immediate change in visual acuity; however, had diplopia on elevation and depression, which improved over six months. Furthermore, she was found to have complete paraesthesia over the right trigeminal distribution. Her vision declined from 20/60 to 20/120 due to keratopathy from the acquired neuropathic cornea.

Patient two; a 52-year-old male was referred due to a recurrence of basal cell carcinoma invading the right medial canthus. The patient underwent robotic-assisted wide local excision and partial rhinectomy, final histology demonstrated complete resection of 10 mm infiltrative basal cell carcinoma with perineural invasion with the nearest margin measuring 4.5 mm (nasal) and 5.5 mm (deep margin). No further adjuvant treatment was required. The residual defect was closed with a composite forehead and right cheek advancement flap.

The patient remains disease-free 18 months post-op with preserved visual function (20/30). The patient has symptomatic epiphora and was referred to the local ophthalmic team for further management.

**Fig. 2:**
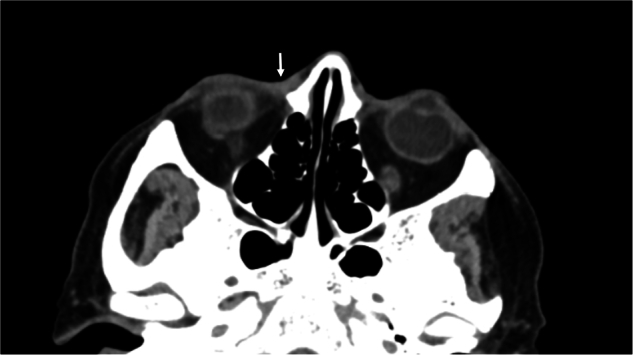
Axial CT image of patient 3. Demonstrating pre-operative right anterior skin thickening with anterior orbital extension (arrow).

**Fig. 3:**
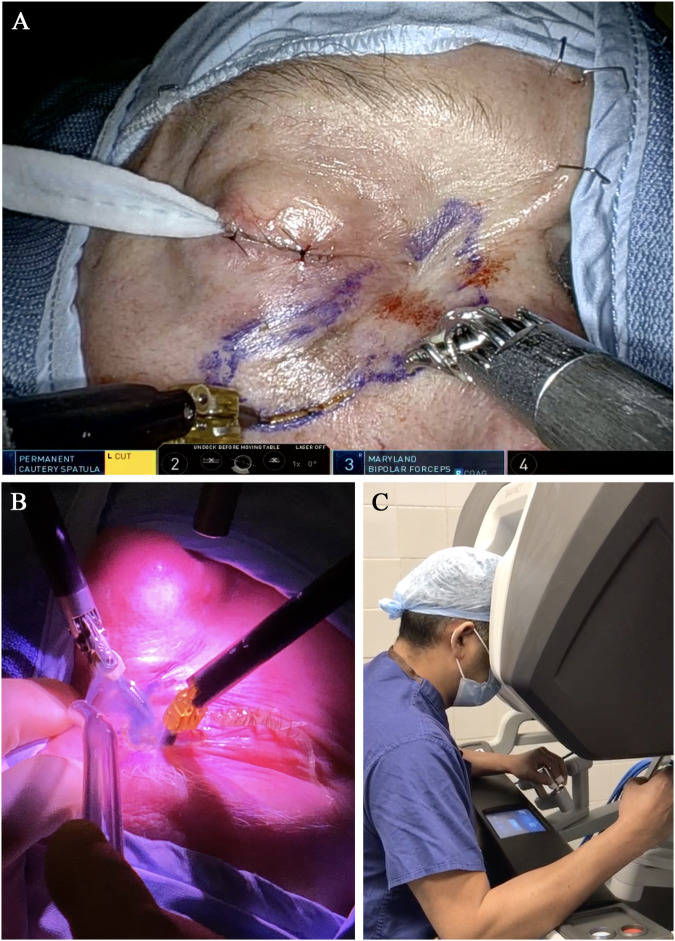
Robotic Assisted Orbital Surgery (RAOS), Comparative operative views. **A** Surgeon view of operative field **B** Bed-side assistant view, **C** Surgical ergonomics and remote console operation.

Patient three; a 70-year-old male presented with a three-year history of epiphora and a right lower lid lesion. Examination demonstrated a medial canthal pigmentary lesion with surface ulceration and telangiectasia, furthermore, on palpation, a firm deep nodule was felt. Imaging demonstrated a small nodular lesion at the medial canthus of the right orbit contiguous with the skin surface (Fig. 2). Biopsy demonstrated two discrete lesions, superficial lentigo maligna and deeper squamous cell carcinoma of the anterior aspect of the lacrimal sac. The patient underwent robotic-assisted wide local excision and partial rhinectomy (Fig. 3; supp video 1) of both lesions. Post-op histology demonstrated complete excision of invasive squamous cell carcinoma of the lacrimal sac (pT1) and lentigo maligna, albeit the latter with a narrow margin (2.5 mm). The patient underwent further resection during the defect reconstruction with a radial forearm free flap on the recommendation of the wider multi-disciplinary team.

Patient three remains disease-free 18 months post-op with excellent visual function (20/15), without reported complications.

The final patient was a 42-year-old male with advanced squamous cell carcinoma of the right medial canthus with a noted pre-auricular lymph node on imaging (Fig. 3). The patient had a history of left-eye optic neuropathy and therefore wanted to preserve vision in the right eye where possible. He underwent robotic-assisted wide local excision with rhinectomy, parotidectomy and neck lymph node clearance. Histology demonstrated narrow margins (inferiorly) with positive lymph nodes, further resection of the inferior margin was performed and the defect was reconstructed with an ulnar-free flap. The patient was recommended to undergo post-operative radiotherapy, however, failed to attend complete planned therapy (total of 20 Gray given).

The patient then developed local recurrence and systemic metastasis four months post-op and subsequently referred for immunotherapy (cemiplimab, PD-1/PDL-1 inhibitor). They developed mild exposure keratopathy post-op which was managed with medical tarsorrhaphy, which improved ocular surface and comfort. The vision was variable due to tear film disturbance and fluctuated between 20/40 and 20/80 (baseline 20/20).

## Discussion

Our pilot study demonstrated the feasibility of robotic assisted orbital surgery applications. We found comparable survival and clinical outcomes to conventional approaches in four challenging cases of advanced periocular tumours.

For advanced tumours invading the orbit, achieving clear margins has shown a survival benefit for all tumour cases [5]. Complete local tumour clearance in such cases is typically achieved through orbital exenteration, a radical clearance of the orbital cavity and contents. This results in marked morbidity from visual loss and disfigurement, which can be particularly life-changing in patients with sight in only one eye. More recently, globe-saving surgery could be achieved in lacrimal gland tumours [6, 7], provided the surgical limits and disease extension are localised to the lateral aspect of the orbit and are easily reachable via open surgical fields. Conversely, tumours arising from medial orbit carry a poor prognosis due to an aggressive invasion path and difficulty in achieving tumour clearance whilst preserving the globe.

Recently, there has been an increased uptake of endoscopic orbital and lacrimal surgery. Early experience with endoscopic approaches demonstrate promising results with novel management approaches to perineural invasion [8] and orphan diseases such as sphenoid wing meningiomas through interdisciplinary working between neuro and ophthalmic surgeons [9–11]. Endoscopes provide a superior intra-operative view compared to standalone microscope, however, remains technically more difficult due to manual operation, instrument crowding, and single-handed operation for tissue dissection. Although, trans-orbital neuro endoscopic surgery (TONES) has overcome this limitation through multi-port access (bi-orbital, trans-nasal). We found robotic surgery confers advantages over the limitation of endoscopic and open surgical approaches, with multi-axial three-dimensional viewing with enhanced visualisation of the operative field, single-port bi-manual operation, tremor elimination and better surgical ergonomics through remote console operation.

The Robotic-assisted Orbital Surgery (RAOS) approach was developed through a multi-disciplinary approach translating experience from Trans-oral robotic-assisted surgery (TORS), which has overcome similar anatomical challenges with a “funnel” effect of anatomically hard boundaries [12, 13]. TORS has demonstrated benefit in oropharyngeal cancers with similar clinical outcomes to conventional treatments, however, improvement in patient-reported outcomes [14, 15], has led to increased recognition and use in other head and neck cancers [16]. Similarly, early experiences are beginning to emerge using DaVinci robotic system for other aspects of orbital surgery, such as thyroid eye disease fat-only decompression [17], and robotic powered endoscopic devices that aid visualisation without limiting single-handed surgery [18].

New devices have preceded many of the major advances in surgical practice. The process of translation of devices from lab to clinical practice can be seen as analogous to the process of drug development, however, contextually different in the developmental process which is reflected by the difference in regulatory environment. Such regulations in the medical device industry have typically lagged behind drug discovery. The IDEAL (idea, development, exploration, assessment, long-term follow) framework, which has proposed a model for device innovation (IDEAL-D) [19], mandates the development of devices to develop and evaluate processes in an ordered and logical manner that balances innovation and safety. Given the current and future robotic devices, there is no single way of evaluating efficacy and safety. In the surgical setting, modification to procedures evolves in a clinical setting. However, reaching first in human study is arguably, the most challenging aspect of the translation of new surgical devices. Direct translation of TORS to RAOS remains limited largely due to several factors:

Firstly, the current physical limitation of the device. The large calibre tools (5–22 mm diameter) that were designed for large cavities such as thorax or abdomen, physically limit further exploration into the significantly small space such as middle or posterior orbit.

Secondarily, lack of haptic feedback necessitates a bedside assistant to protect soft tissue and the globe from an indirect crush injury or fracture.

Thirdly, much of current orbital practice involves bone remodelling (decompression) which is not possible with currently available tools.

Finally, the impact of biomechanical stretch and electro-thermal effects on orbital tissue remains unknown. Further pre-clinical development and refinement of tools to assess the efficacy and safety of such tools around electro-thermal sensitive tissues like the eye is therefore required before wider use in non-sight or life-threatening conditions.

Our pilot study demonstrates the potential benefits and feasibility of robotic-assisted surgical devices in tumour surgery in this anatomically challenging area. Current limitations, therefore, necessitate further development before wider implementation through reverse engineering of current available robotic assisted surgical systems for adnexal applications or through bespoke development, which may benefit allied specialities that require finer instrumentation for smaller cavities, such as paediatrics, otologist or neurosurgeons.

## Conclusion

Our series demonstrates the potential benefits of a widely used robotic surgical system for tumour clearance in the anterior orbit. The advantages of three-dimensional optics, multi-directional instrumentation, motion scaling technology for tremor elimination, and improved surgical ergonomics may contribute to the further development of minimal access orbital and trans-orbital surgery. However, additional work is warranted to evaluate safety, develop robotic instrumentation for deep socket exploration, address common orbital procedures such as orbital decompression, and determine patient benefits.

## Summary

### What was known before

- Robotic-assisted surgical systems have been widely adopted in various surgical fields over the past 20 years due to their enhanced precision, manoeuvrability, and visualisation capabilities.
- Randomised controlled trials have shown that robotic-assisted procedures offer comparable clinical outcomes to traditional surgery, with potential benefits including improved quality of life, quicker patient recovery.
- Robotic-assisted surgical devices enable scaled motion and enhanced visualisation, making them a promising tool for procedures in this delicate and challenging anatomical area.

### What this study adds

- The pilot study on robotic-assisted orbital surgery demonstrated the feasibility of using robotic systems for challenging cases of advanced periocular tumours.
- The size of current robotic tools limits exploration into significantly smaller spaces, such as the middle or posterior orbit.
- Our study highlights the need for structured development and evaluation processes for surgical devices to ensure innovation while maintaining safety.

### Supplementary information

eye-reporting-checklist.pdf

Robotic Assisted Orbital Surgery Resection of Advanced Periocular Tumours Case 3

Video Legend

## Data Availability

A minimal data set of outcomes has been included in the manuscript.
