# Supplementary material for: Robotic assisted orbital surgery for resection of advanced periocular tumours – a case series report on the feasibility, safety and outcome
Source: Eye (Lond). 2024 Feb 22;38(8):1496–501. doi: 10.1038/s41433-024-02932-6 (PMC11126675; doi:10.1038/s41433-024-02932-6)
Supplement: Supplementary file 3 — Video Legend [file 41433_2024_2932_MOESM3_ESM.docx]

**Supplemental Material**

Video illustration of robotic-assisted device set up, operating room layout and operative view.
